# Assessing law enforcement officer skills in Crisis Intervention Team (CIT) research: developing and implementing standardized scenarios

**DOI:** 10.3389/fpsyg.2024.1463462

**Published:** 2024-12-17

**Authors:** Amy C. Watson, Elisabeth Jackson, En Fu, Ron Bruno, Erin Comartin, Don Kamin, Leah G. Pope, Eduardo Vega, Michael T. Compton

**Affiliations:** ^1^School of Social Work, Wayne State University, Detroit, MI, United States; ^2^Department of Psychiatry, Columbia University Vagelos College of Physicians and Surgeons, New York, NY, United States; ^3^Crisis Response Programs and Training, Inc., Salt Lake City, UT, United States; ^4^Institute for Police, Mental Health & Community Collaboration at Coordinated Care Services, Inc., Rochester, NY, United States; ^5^New York State Psychiatric Institute, New York, NY, United States; ^6^Humannovations, Pasadena, CA, United States

**Keywords:** Crisis Intervention Team, law enforcement, police, mental health crisis, standardized scenarios

## Abstract

This paper outlines the development of standardized scenarios used to assess the efficacy of Crisis Intervention Team (CIT) training in a randomized, controlled trial. The objective was to create scenarios that accurately simulate mental health crisis situations for law enforcement officers, ensuring that each scenario tests specific CIT skills relevant to real-world encounters. Our process involved building an interdisciplinary team and drawing from the knowledge and experience of professionals in law enforcement, mental health, and performance arts to design a set of scenarios that are both realistic and challenging. The scenarios were developed to represent mental health crises that officers frequently encounter, such as mania, psychosis with agitation, and depression with suicidality. Each scenario requires officers to demonstrate CIT-specific skills such as verbal de-escalation, empathy, and critical decision-making. Our structured approach to developing these scenarios provides a replicable model for future studies that aim to assess the real-world effectiveness of mental health training for law enforcement as well as other professional groups.

## Introduction

The Crisis Intervention Team (CIT) model was first developed in 1988 in Memphis, Tennessee—after a fatal police shooting and subsequent public outcry—to improve how police officers respond to people experiencing mental health crises. The model, based on community partnerships, includes a 40-h training for police officers delivered by law enforcement trainers, mental health professionals, and people with mental illnesses and their family members. Much of the CIT training week focuses on teaching de-escalation and crisis response skills, with approximately nine of the 40 h of the training devoted to scenario-based role-play providing officers with the opportunity to practice and demonstrate that they have acquired the skills needed to safely and effectively respond to people experiencing mental health crises.

CIT training is considered evidence-based for improving officer-level outcomes (Veluri and Mansuri, 2021; Watson et al., 2017). Specifically, CIT-trained officers exhibit greater knowledge, improved attitudes, reduced stigma, greater self-efficacy, and better decision-making regarding mental health-related encounters (Compton et al., 2014a; Kubiak et al., 2017). There is also strong evidence that CIT model implementation and training increases linkages to mental health services (Bratina et al., 2020; Compton et al., 2014b; Kubiak et al., 2017; Watson et al., 2021), especially when officers self-select into the specialist role (Compton et al., 2017) and in areas with ample mental health resources (Watson et al., 2011). The evidence on CIT training’s impact on other important outcomes such as arrests and use of force is mixed, with some studies finding no statistically significant effects and other studies finding effects under specific conditions (Compton et al., 2014b; Morabito et al., 2012; Willis et al., 2023). Conducting research on events such as arrests, use of force, injuries, and deaths is complicated by varied definitions and documentation practices, as well as the need for samples of mental health-related police contacts large enough to detect changes in relatively low-frequency events. While larger agencies may respond to enough mental health-related calls, their data systems typically are not designed to allow for reliable identification of mental health-related encounters suitable for research purposes.

No studies have examined the effectiveness of CIT training for improving officers’ actual skills when responding to mental health crises. Field data—such as field observations or body-worn camera footage—could be used to examine officers’ skills performance; however, it does not allow researchers to control the situations to which officers are exposed and responding. Further, body-worn camera footage provides limited views of the encounter insofar as it is focused on the subject in crisis rather than the officer and his or her behavior; its use in research also presents sensitive human subjects protections issues.

Given the limited research on the effect of CIT training on officers’ skills in responding to people experiencing a mental health crisis, as well as the challenges of using field data to study such outcomes, we designed a multi-site, randomized, controlled trial of CIT training using standardized role-play scenarios to measure officers’ skills. Such an approach allows the research team to strictly control what CIT-trained and non-CIT-trained participating officers are exposed to and to measure skills performance in a high-fidelity and controlled manner. In this research note, we describe our approach to developing and implementing three sets of standardized scenarios (nine in total). The primary outcome of interest is demonstrated skills observed among officers randomized to CIT training or to a control group, measured at baseline and 3 and 6 months post-training.

### The use of standardized scenarios and simulations for training

Standardized scenarios and training simulations are extensively utilized for training, assessing, and predicting real-world performance in various professions, including airline piloting, air traffic controlling, social work, pharmacy, medicine, and policing, among others. Like pilot training, air traffic control relies heavily on simulations to train controllers by implementing realistic, high-pressure experiences to improve decision-making skills and response times (Robinson et al., 2017). In social work, interactive simulations are used to train professionals in client communication and enable them to practice sensitive conversation skills in controlled settings (Olson et al., 2015). Pharmacy education incorporates simulations to teach students how to handle real-life patient interactions and facilitates a better understanding of patient care, improving communication skills and enabling students to practice managing complex situations (Kerr et al., 2021); medical specialties also use simulation labs and “standardized patients” who may be actors. Similarly, law enforcement agencies utilize simulation to train officers in decision-making and interpersonal interactions, with scenarios often focusing on crisis intervention and use-of-force decisions (Lavoie et al., 2023). LaVoie et al. (2022) describe the co-creation of mental health crisis response training for police officers and the use of scenario-based Forum Theater methods for building and assessing core competencies. In discussing police training in procedural justice, Rosenbaum and Lawrence (2017) emphasize the importance of experiential practice in the form of scenarios and role playing, repetition, and immediate feedback for skill mastery, while also noting that the transfer of learning to behavior on the job is challenged by socialization processes favoring toughness, as well as officer safety and agency culture.

While simulation environments are sometimes criticized for their artificiality, studies consistently show a significant positive correlation between simulation and actual performance. In the field of airline piloting, for example, high-fidelity simulators are critical for training pilots on complex maneuvers and emergency scenarios, significantly enhancing flight safety (Myers et al., 2018). Yang et al. (2019) demonstrated the efficacy of high-fidelity simulation in Intensive Care Unit residency training as an assessment tool, showing a significant correlation between simulation and actual clinical performance. Similarly, Weersink et al. (2019) found that resuscitation simulation performance among emergency medicine residents closely mirrored their performance in real clinical settings.

Standardized scenarios and simulations take several forms, but typically include scripting and training role-players to respond to trainees based on their behaviors, attitudes, and verbal communication (Rosenbaum and Lawrence, 2017). The use of standardized scenarios in research on the effectiveness of training programs typically involves actors that are carefully trained to portray specific mental or physical states to ensure consistency across role-play scenarios (Cross et al., 2010). These role-play scenarios are recorded and subsequently rated, using observational rating scales (Cross et al., 2007, 2010). This approach has previously been used, though rarely, in research on police training. For example, Rosenbaum and Lawrence (2017) used standardized role-play scenarios and observed and coded recruits’ skills to evaluate the effectiveness of training on procedurally just policing.

## Approach

### Selecting scenario topics

The mental health conditions and situations portrayed in our study scenarios were selected to represent common types of mental health crisis calls that officers respond to and maximize opportunities for participants to demonstrate CIT-specific skills. To create an initial list of candidate conditions and call situations, we drew from our prior work examining police encounters with people with serious mental illnesses, which has included conducting ride-along observations and interviews with police officers, analyzing data on mental health-related calls for service, and interviewing people with serious mental illnesses about their encounters with police (Compton et al., 2014b; Watson et al., 2008; Watson et al., 2021; Wood et al., 2021; Wood et al., 2017). We also reviewed CIT training scenarios utilized by two statewide CIT programs and multiple local CIT programs. We discussed this list among the research team, including our law enforcement advisors, and selected three mental health crisis presentations for inclusion that would allow participants to demonstrate the range of skills taught in CIT training. Each mental health crisis presentation would have three different versions, with each version to be portrayed by a single actor. Thus, early on we eliminated scenarios that would require officers to interact “on scene” with family members or bystanders in addition to the primary person in crisis.

Prior to developing the scenarios, we conducted a literature search on de-escalation and crisis intervention skills for law enforcement, mental health, and other professionals. We reviewed standard CIT training curricula and skills checklists used by CIT programs. We also reviewed literature and training related to procedural justice. This work informed scenario development and provided the basis for our scenario skills coding tool (to be described in another report). Specifically, for each mental health crisis presentation, we made a list of the CIT skills that officers might utilize. Scenarios were developed to challenge officers and provide opportunities for demonstration of the identified CIT skills, with three overarching presentations:

- Mania and Refusal to Leave. This scenario involves a subject who is very “revved up” working on a project at a location where they have been asked to leave. The subject is insistent that what they are working on is very important. Their speech is rapid and tangential, and their elation shifts to irritation with those who do not understand the importance of what they are doing. Police have been called to get them to leave.
- Psychosis with Agitation. This scenario involves a subject experiencing auditory hallucinations and delusions. The subject is agitated, talking to themself, responding to internal voices, and holding a blunt object. Police have been called because the subject is on private property creating a disturbance and alarming people.
- Depression with Suicidality. This scenario involves a subject who is depressed, has recently experienced losses (e.g., ending of a relationship, losing a job) and has shown indications of suicidality. The subject presents as hopeless, sad, and remorseful. The subject has a history of a prior suicide attempt. Police have been asked to check on them.

We developed three versions of each scenario (nine in total) so that officers are not exposed to the exact same scenario more than once across the three testing points in the randomized trial, and to reduce practice effects. Within each scenario set, we varied the settings and details of the situation, but maintained consistency in terms of symptom acuity, level of risk, and legal factors (potential for arrest).

Both the mania and psychosis scenarios were intentionally constructed as situations that officers could choose to interpret as including an arrestable offense (e.g., criminal trespass) and that could lead to the use physical force (e.g., for apprehension and removal). The mania scenario was designed to challenge officers to demonstrate empathy, patience, and de-escalation techniques. The presence of agitation and a blunt weapon in the psychosis scenario increased the intensity and risk involved in the scenario to provide officers an opportunity to demonstrate the use of time, space, and cover along with verbal de-escalation skills. The depression with suicidality scenario provided opportunities for officers to demonstrate empathy, active listening skills, and the ability to effectively ask suicide-related questions. The scenarios were designed to be portrayed by actors of any gender or race. For example, mania and refusing to leave a public library can be cast: as a black female, a white female, a black male, a white male, etc. For our study, we cast the role for the psychosis scenario as a Black male, the role for the mania scenario as a Black female, and the role of the depression/suicidality scenario as a White male. Practical limitations prevented us from varying race and gender within scenario sets, as that would have required a larger number of actors and such varying would interfere with the goal of completely standardizing the officer’s exposure at baseline, 3-months, and 6-months. Given the three scenario types, we were also not able to balance race and gender variation across scenario sets. However, we intentionally selected the race and gender of the person in crisis across the scenario sets to be sure we were not simply testing whether CIT “works” for improving response to one demographic group.

### Creating three versions of each scenario topic

As noted, the study design includes baseline, 3-month, and 6-month testing of participants. At each time point, officers complete three standardized scenarios, one for each of the three selected mental health crisis presentations. This required ensuring that the three versions of each scenario presentation were different enough in terms of setting and story, while also: (1) being of an equal level of “difficulty” for the officer (including equal safety risks and potential for arrest), (2) allowing for the demonstration of the same CIT-specific skills in each version, and (3) being suitable for the same actor to perform each version. Drawing from work of Cross et al. (2010), we structured the scenario scripts to include the following (see Table 1 for an excerpt): a brief description of the scenario; the information that is provided to the officer prior to arrival on scene (i.e., prior to entering the scenario); scene set-up and props; a scenario summary; the subject’s name and demographic information; the subject’s backstory; the initial scene opening as the officer arrives on scene; specific instructions for the actor for the start, middle, and end of the scenario that include specific behaviors and statements to be displayed by the actor; a statement on the quality and intensity of the subject’s behavior; and directions for increasing or decreasing agitation/intensity based on the officer’s response. Each scenario was designed to be portrayed by the actor for up to 5 min of interaction with the officer.

**Table 1:** Abbreviated scenario script excerpt.

Scenario 1a. Disturbance involving a subject who is hearing voices, paranoid, delusional, and has a blunt-force weapon but is not threatening anyone with it	
Information provided to the officer	You have been dispatched to a transient problem. Caller indicates subject is acting strangely and loitering in the courtyard of an office building where workers like to spend their breaks. Several people have indicated he is making them feel nervous. The caller greets you when you arrive and points you to the door that leads to the courtyard area. He states that this is private property, and he wants the subject removed.
Scene set-up	Office park courtyard, a picnic table (or a table) with space around it on all sides. Subject has a blanket and has slept in the courtyard. Subject is somewhat disheveled and dressed in several layers.
Scenario summary	No one other than the subject is in the picnic area. The subject is agitated, he is pacing, talking loudly to voices, and waving his arms around. He has a pipe in one hand but is not directly threatening himself or anyone else. Subject will comply eventually and do what the officer instructs if the officer speaks calmly to him. If an officer is not calm and respectful (e.g., yells commands, does not introduce self) and does not use verbal de-escalation skills, the subject will remain agitated and only minimally comply.
Details	Steve Booker, DOB 2/17/1983, Male, Black
Subject’s backstory	You struggle with paranoia and voices, and you are sure the government is listening to your thoughts. You had been staying at a homeless shelter nearby. In the past few weeks, you have started suspecting that the staff there are helping the government spy on you, and yesterday, you decided you had to leave. You hear voices that have been getting louder and making it hard for you to think straight, so you need to get outside and away from the surveillance. Last night you slept in the courtyard area of the office park. In the morning, when people started arriving for work, you became convinced the government spies had found you and became very agitated and unsure of what to do next. You have not been in touch with your family for some time, though you have a brother, Mark, who lives with his family nearby. The last time you talked to him was about a year ago when he tried to help you get a job. Your parents are deceased.
Quality/Intensity	You are agitated, paranoid, and distracted by voices. You are fearful that the government will find you and you do not know what to do or where you can go. You do not know if you can trust the officer.
Subject’s behaviors/Statements	Throughout: pacing, responding to voices and waving hands around.
	**START.** You have a pipe in your hand when the officer arrives, **but do not threaten to harm the officer at any time**. If the officer yells at you to drop the pipe, do not drop the pipe—just continue pacing and responding to voices, as described above—by saying “Shut the fuck up, stop it, I need to think…” When saying “shut the fuck up,” you are not looking at the officer, but rather are looking to the side and at times you put your head in your hands while saying “let me think, let me fucking think.” If the officer calmly asks you to put down the pipe, you comply slowly and set it on the ground. Set it right at your feet. If the officer calmly asks you to move away from it, do so. If the officer yells instructions, do not move away—make them work for it and ask calmly—take your time but do it. If the officer unholsters their gun or points their gun at you, ratchet up the escalation. (You can still eventually comply with commands if the officer speaks calmly to you with gun out).

In constructing the psychosis and mania scenarios, we intentionally created situations in which an officer might choose to use force to gain control of the subject, but even at the highest level of intensity would not present a level of threat to the officer that would require the use of force. Specifically, while the subject in the psychosis scenario was agitated and holding a blunt object that could be used as a weapon (a pipe or a wrench), when the scenario begins, he is more than 20 feet away from the arriving officer and he never closes distance or raises the weapon in a threatening manner. Likewise, the subject experiencing mania never closes distance on the officer; rather, they moves away if the officer moves toward her.

### Vetting and finalizing the nine standardized scenarios

The study investigators have extensive experience working and conducting research in mental health and criminal legal settings. However, we felt that to ensure the scenarios would be believable and relevant to police officers and accurate in terms of portrayal of crisis states officers encounter, it was imperative that we engage law enforcement and clinical content experts to assist us. Our law enforcement experts provided us insight on what typical mental health calls look like in the field, as well as how police officers might respond to crisis-related behaviors and situations. They provided guidance on what officers look for on a call scene, specific tactics they are trained in, and what behaviors are likely to elicit uses of force. This allowed us to structure scenarios to provide opportunities for officers to demonstrate CIT-related skills. To that end, we drafted adaptable, dynamic scenario scripts that would allow the officer to influence the trajectory of the scene, whether that meant they utilized de-escalation skills, relied on their authority and control tactics, or something in between.

Similarly, it was important that the scenarios capture the accurate presentations of the three mental states. For this, we consulted clinical experts—a clinical forensic psychologist, a clinical psychologist, and a psychiatrist. They complemented the police perspective with a clear picture of how a person may experience symptoms of psychosis, mania, or depression/suicidality and how they might react, in a moment of crisis, to law enforcement. The aid of theater performance consultants was enlisted to help us craft scenarios with clear instructions for the actors on their role and the boundaries within which they were to improvise based on the quality of the officer’s interaction. Our theater experts also helped us to hire and train actors who had both the stamina and the range to perform the scenario roles accurately and consistently up to 45 times in one testing day.

Concurrently with scenario development, we were developing the rating scale to be used to rate the recorded videos in which officers demonstrate the skills of interest to varying degrees. Recurrent collaborative sessions were held with the content experts to discuss what participant actions and verbal communications were likely to be, or should be, elicited by the different scenarios and how the rating scale could capture variability in officer performance. Development of the rating scale is reported on separately.

Once we were confident that we had complete near-final drafts of the scenarios, we asked our content experts and several external law enforcement advisors for feedback on equivalence across each of the three scenario versions of each mental health crisis scenario type, as well as the degree to which they would feel realistic to officers and modified the scenarios accordingly. For example, we changed the location of one of the mania scenario versions so that all three took place on public property and changed wording on one of the psychosis scenarios so that all three clearly took place on private property. An abbreviated example of a scenario is provided in Table 1. Our approach to incorporating expertise from multiple fields helped us ensure that the scenarios were realistic; provided opportunities for officers to display CIT-related skills; were equivalent across the three versions of each mental health condition in terms of difficulty, risk, and legal considerations; and provided actors with adequate guidance on how to perform their roles.

## Discussion

The process that we followed resulted in nine highly standardized scenarios that we are using as the main stimuli/exposures for collecting primary outcome data for a randomized, controlled trial involving law enforcement officers’ demonstrated crisis intervention skills. The exposure/experience is not dissimilar to simulations used in other fields for both training and research purposes. Future reports will describe the development and psychometric properties of a standardized scoring procedure for rating video-recordings of officers’ interactions during these standardized scenarios (as a means of data collection for primary and secondary outcomes), as well as our experience with identifying, auditioning, training, employing, and rehearsing professional actors to perform the mental health crisis scenarios.

One of the primary lessons learned was the value of interdisciplinary collaboration in creating highly realistic and standardized scenarios. Clinicians, experienced law enforcement professionals, researchers with experience utilizing written and video vignette methodologies, and a theater expert (in both performance and directing) worked together to ensure that each scenario was authentic, relevant, and consistent. Additionally, clear safety instructions, developed in collaboration with our law enforcement team members and advisors, provided actors with the ability to safely engage with officers in realistic scenarios. The development process for the scenario script framework, the specific scenarios’ contexts, and actor instructions was iterative. We worked with one set of scenarios (e.g., psychosis with agitation) at a time, but on several occasions made decisions related to formatting and instructions while working on one set that we then applied to the others.

Compared to other approaches to measuring officers’ skills, such as field observations and body-worn camera recordings, our standardized scenarios enable us to isolate and evaluate specific officer behaviors and decisions in a controlled environment. This allows us to control precisely what officers are exposed to, accurately measure officers’ ability to demonstrate skills, and assess the impact of CIT training on those skills.

Despite the strengths of our approach, there are several limitations that must be acknowledged. The practical considerations of our approach limited us to three mental health crisis presentations, each of which had three versions that could be run with a single actor (i.e., three actors), which consequently excluded other frequent mental health crises encountered by officers, such as calls to family-related conflicts between a caregiver and a symptomatic loved one. However, we feel that across the three scenario types, officers are provided with ample opportunities to demonstrate key CIT-related skills. While the scenarios can be cast for actors of any demographic groups, practical considerations do not allow us to vary the race/ethnicity and gender of the scenario subject (actor) and subsequently examine any variation in officers’ use of CIT skills by the subject’s or the officer’s demographic variables. Because we cannot practically implement within-scenario-set variation of the subject’s demographics, we intentionally implemented between-scenario-set variation of gender and race. Further, we cast the two scenario presentations with the highest potential for use of force and arrest as Black actors, given known disparities across the criminal legal system. While we are not able to measure or control for the impact of racial bias, if we do find a CIT effect on officers’ skill demonstration as measured by their scenario performance, it will not be limited to responses to White subjects. Finally, we must acknowledge the limitations of using standardized role-play scenarios, regardless of how realistic, as an approach to measuring the effectiveness of CIT training. While research from other fields has found performance in standardized scenarios to be predictive of on-the-job performance (Weersink et al., 2019; Yang et al., 2019), we are not measuring performance in the field. As Rosenbaum and Lawrence (2017) note, law enforcement culture may impede the transfer of skill learning to performance in the field. Our efforts to incorporate law enforcement, clinical, and theatrical expertise supported the development of highly realistic scenario experiences for the officers. We hope this increases the extent to which officer scenario performance reflects their real-world performance when responding to mental health-related calls. That is a question for future research.

## Conclusion

Our use of standardized scenarios in a randomized, controlled trial examining the effectiveness of CIT training for improving officers’ skills represents an important but underutilized approach in police training research. Our rigorous approach to scenario development—designed to portray realistic and challenging mental health crisis situations—enables a detailed assessment of officers’ CIT-related skills.

## Author’s note

Once data collection for this study has been completed, full scenario scripts and scoring procedures will be made publicly available.

## References

[ref1] Bratina M. P. Carrero K. M. Kim B. Merlo A. V. (2020). Crisis intervention team training: when police encounter persons with mental illness. Police Pract. Res. Int. J. 21, 279–296. doi: 10.1080/15614263.2018.1484290

[ref2] Compton M. T. Bakeman R. Broussard B. D’Orio B. Watson A. C. (2017). Police officers’ volunteering for (rather than being assigned to) crisis intervention team (CIT) training: evidence for a beneficial self-selection effect. Behav. Sci. Law 35, 470–479. doi: 10.1002/bsl.2301, PMID: 28940465 PMC5741493

[ref3] Compton M. T. Bakeman R. Broussard B. Hankerson-Dyson D. Husbands L. Krishan S. . (2014a). The police-based crisis intervention team (CIT) model: I. Effects on officers’ knowledge, attitudes, and skills. Psychiatr. Serv. 65, 517–522. doi: 10.1176/appi.ps.201300107, PMID: 24382628 PMC11420632

[ref4] Compton M. T. Bakeman R. Broussard B. Hankerson-Dyson D. Husbands L. Krishan S. . (2014b). The police-based crisis intervention team (CIT) model: II. Effects on level of force and resolution, referral, and arrest. Psychiatr. Serv. 65, 523–529. doi: 10.1176/appi.ps.201300108, PMID: 24382643

[ref5] Cross W. Matthieu M. M. Cerel J. Knox K. L. (2007). Proximate outcomes of gatekeeper training for suicide prevention in the workplace. Suicide Life Threat. Behav. 37, 659–670. doi: 10.1521/suli.2007.37.6.659, PMID: 18275372

[ref6] Cross W. Matthieu M. M. Lezine D. Knox K. L. (2010). Does a brief suicide prevention gatekeeper training program enhance observed skills? Crisis 31, 149–159. doi: 10.1027/0227-5910/a000014, PMID: 20573609 PMC2913886

[ref7] Kerr A. Strawbridge J. Kelleher C. Barlow J. Sullivan C. Pawlikowska T. (2021). A realist evaluation exploring simulated patient role-play in pharmacist undergraduate communication training. BMC Med. Educ. 21:325. doi: 10.1186/s12909-021-02776-8, PMID: 34092216 PMC8180382

[ref8] Kubiak S. Comartin E. Milanovic E. Bybee D. Tillander E. Rabaut C. . (2017). Countywide implementation of crisis intervention teams: multiple methods, measures and sustained outcomes. Behav. Sci. Law 35, 456–469. doi: 10.1002/bsl.2305, PMID: 28983959

[ref9] Lavoie J. Álvarez N. Baker V. Kohl J. (2023). Training police to de-escalate mental health crisis situations: comparing virtual reality and live-action scenario-based approaches. Policing 17:paad069. doi: 10.1093/police/paad069

[ref10] Lavoie J. Alvarez N. Kandil Y. (2022). Developing community co-designed scenario-based training for police mental health crisis response: a relational policing approach to De-escalation. J. Police Crim. Psychol. 37, 587–601. doi: 10.1007/s11896-022-09500-2, PMID: 35250163 PMC8882363

[ref11] Morabito M. S. Kerr A. N. Watson A. Draine J. Ottati V. Angell B. (2012). Crisis intervention teams and people with mental illness. Crime Delinq. 58, 57–77. doi: 10.1177/0011128710372456

[ref12] Myers P. Starr A. Mullins K. (2018). Flight simulator fidelity, training transfer, and the role of instructors in optimizing learning. Int. J. Aviat. Aeronaut. Aerosp. 5, 1–26. doi: 10.15394/ijaaa.2018.1203

[ref13] Olson M. D. Lewis M. Rappe P. Hartley S. (2015). Innovations in social work training: a pilot study of interprofessional collaboration using standardized clients. Int. J. Teach. Learn. High. Educ. 27, 14–24.

[ref14] Robinson J. E. Lee A. Lai C. F. (2017). “Development of a high-fidelity simulation environment for shadow-mode assessments of air traffic concepts” in Aeronautical society modeling and simulation in air traffic management conference (London). Available at: https://ntrs.nasa.gov/citations/20170011122 (Accessed November 26, 2024).

[ref15] Rosenbaum D. P. Lawrence D. S. (2017). Teaching procedural justice and communication skills during police-community encounters: results of a randomized control trial with police recruits. J. Exp. Criminol. 13, 293–319. doi: 10.1007/s11292-017-9293-3

[ref16] Veluri N. Mansuri Z. (2021). Does the crisis intervention team (CIT) training improve police officers’ knowledge, attitude, and mental health stigma? Eur. Psychiatry 64, S466–S467. doi: 10.1192/j.eurpsy.2021.1246

[ref17] Watson A. C. Angell B. Morabito M. S. Robinson N. (2008). Defying negative expectations: dimensions of fair and respectful treatment by police officers as perceived by people with mental illness. Adm. Policy Ment. Health Ment. Health Serv. Res. 35, 449–457. doi: 10.1007/s10488-008-0188-5 18661226

[ref18] Watson A. C. Compton M. T. Draine J. N. (2017). The crisis intervention team (CIT) model: an evidence-based policing practice? Behav. Sci. Law 35, 431–441. doi: 10.1002/bsl.2304, PMID: 28856706

[ref19] Watson A. C. Ottati V. C. Draine J. Morabito M. (2011). CIT in context: the impact of mental health resource availability and district saturation on call dispositions. Int. J. Law Psychiatry 34, 287–294. doi: 10.1016/j.ijlp.2011.07.008, PMID: 21820177 PMC3171588

[ref20] Watson A. C. Owens L. K. Wood J. Compton M. T. (2021). The impact of crisis intervention team response, dispatch coding, and location on the outcomes of police encounters with individuals with mental illnesses in Chicago. Policing 15, 1948–1962. doi: 10.1093/police/paab010, PMID: 34659453 PMC8507917

[ref21] Weersink K. Hall A. K. Rich J. Szulewski A. Dagnone J. D. (2019). Simulation versus real-world performance: A direct comparison of emergency medicine resident resuscitation entrustment scoring. Adv. Simul. 4:9. doi: 10.1186/s41077-019-0099-4, PMID: 31061721 PMC6492388

[ref22] Willis T. Kern L. J. Hedden B. J. Nelson V. Comartin E. Kubiak S. (2023). The impact of crisis intervention team (CIT) training on police use of force. J. Offender Rehabil. 62, 157–173. doi: 10.1080/10509674.2023.2182863

[ref23] Wood J. D. Watson A. C. Barber C. (2021). What can we expect of police in the face of deficient mental health systems? Qualitative insights from Chicago police officers. J. Psychiatr. Ment. Health Nurs. 28, 28–42. doi: 10.1111/jpm.12691, PMID: 32966680

[ref24] Wood J. D. Watson A. C. Fulambarker A. J. (2017). The “gray zone” of police work during mental health encounters: findings from an observational study in Chicago. Police Q. 20, 81–105. doi: 10.1177/1098611116658875, PMID: 28286406 PMC5342894

[ref25] Yang C.-W. Ku S.-C. Ma M. H.-M. Chu T.-S. Chang S.-C. (2019). Application of high- fidelity simulation in critical care residency training as an effective learning, assessment, and prediction tool for clinical performance. J. Formos. Med. Assoc. 118, 1347–1355. doi: 10.1016/j.jfma.2018.12.003 30584004

